# QuickStats

**Published:** 2014-12-05

**Authors:** FROM THE NATIONAL CENTER FOR HEALTH STATISTICS

**Figure:**
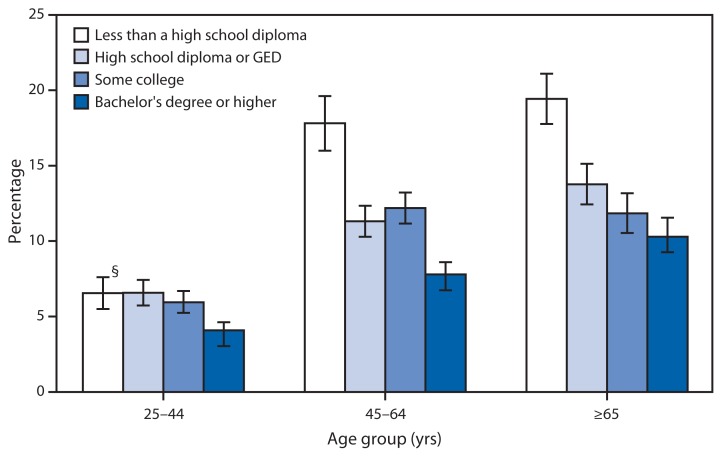
Percentage of Adults Aged ≥25 Years with Trouble Seeing When Wearing Corrective Lenses,* by Education Level and Age Group — National Health Interview Survey, United States, 2012–2013^†^ **Abbreviation:** GED = General Educational Development certificate. * Based on response to the question, “Do you have trouble seeing, even when wearing glasses or contact lenses?” ^†^ Estimates are based on household interviews of a sample of the noninstitutionalized U.S. civilian population and are derived from the National Health Interview Survey sample adult component. ^§^ 95% confidence interval.

The percentage of adults who reported having trouble seeing when wearing corrective lenses declined with education level for all age groups. Among adults aged 25–44 years in 2012–2013, 6.6% of those who did not graduate from high school reported trouble seeing, compared with 4.1% of those who had a bachelor’s degree or higher. Trouble seeing was about twice as likely for those who did not graduate from high school compared with those with a bachelor’s degree or higher among adults aged 45–64 years (17.8% versus 7.8%) and aged ≥65 years (19.4% versus 10.3%). The percentage of adults aged 45–64 years and ≥65 years who reported trouble seeing was higher than the percentage for adults aged 25–44 years at every level of education.

**Source:** National Health Interview Survey, 2012–2013. Available at http://www.cdc.gov/nchs/nhis.htm.

**Reported by:** Brandy Lipton, PhD, xlw7@cdc.gov, 301-458-4318; Sandra Decker, PhD.

